# Immature, Semi-Mature, and Fully Mature Dendritic Cells: Toward a DC-Cancer Cells Interface That Augments Anticancer Immunity

**DOI:** 10.3389/fimmu.2013.00438

**Published:** 2013-12-11

**Authors:** Aleksandra M. Dudek, Shaun Martin, Abhishek D. Garg, Patrizia Agostinis

**Affiliations:** ^1^Laboratory of Cell Death Research and Therapy, Department of Cellular and Molecular Medicine, KU Leuven, Leuven, Belgium

**Keywords:** immunogenic cell death, phenotypic DC maturation, cytokine, antigen, cell death, cancer, immunosurveillance, chemotherapy

## Abstract

Dendritic cells (DCs) are the sentinel antigen-presenting cells of the immune system; such that their productive interface with the dying cancer cells is crucial for proper communication of the “non-self” status of cancer cells to the adaptive immune system. Efficiency and the ultimate success of such a communication hinges upon the maturation status of the DCs, attained following their interaction with cancer cells. Immature DCs facilitate tolerance toward cancer cells (observed for many apoptotic inducers) while fully mature DCs can strongly promote anticancer immunity if they secrete the correct combinations of cytokines [observed when DCs interact with cancer cells undergoing immunogenic cell death (ICD)]. However, an intermediate population of DC maturation, called semi-mature DCs exists, which can potentiate either tolerogenicity or pro-tumorigenic responses (as happens in the case of certain chemotherapeutics and agents exerting ambivalent immune reactions). Specific combinations of DC phenotypic markers, DC-derived cytokines/chemokines, dying cancer cell-derived danger signals, and other less characterized entities (e.g., exosomes) can define the nature and evolution of the DC maturation state. In the present review, we discuss these different maturation states of DCs, how they might be attained and which anticancer agents or cell death modalities (e.g., tolerogenic cell death vs. ICD) may regulate these states.

## Introduction

It is conceptually established that the immune system can be distributed across two basic components, i.e., the innate immune system and the adaptive immune system (1, 2). The primary aim of innate immune cells is to provide a rapid non-specific response to any pathogen or foreign aggressors (possessing foreign antigens), wound, inflammatory insult, or newly initiated diseased cell (owning possible “non-self” antigens) (1, 2). On the other hand, the primary aim of adaptive immune cells is to provide a latent but highly specific response against foreign or “non-self” antigens and to generate an “immune memory” against these antigens to counter similar insults in the future more quickly (either cell interaction dependent or independent; the latter applying to antibody production) (3, 4). Together these two branches of the immune system are supposed to initiate acute inflammation ultimately culminating in its resolution and wound healing once they have taken care of the aggressor, insult, or diseased cell (5, 6). It is noteworthy that in terms of evolution, the conception of the innate immune system pre-dates that of the adaptive immune system (1). Most notable innate immune cells include macrophages, natural killer (NK) cells, dendritic cells (DCs), various myeloid lineage subsets, neutrophils, basophils, and eosinophils (1, 6); while the most notable adaptive immune cells include T and B lymphocytes (3, 5).

The initial reaction orchestrated by innate immune cells consists of capturing, as well as clearing up or destroying the source of injury, infection, or diseased cells, followed by wound healing and if required (in case of well discernable antigens) “priming” of the adaptive immune cells against antigens derived from the “non-self” diseased cells or pathogens (1, 2). This adaptive immune cell priming helps to initiate more specific responses, directed against the acquired antigens and leading to the eradication of the antigen source (3, 6). This in principle is also the basic theory behind anticancer immunity or anticancer immunosurveillance (7), where innate immune cells recognize the “non-self” tumor-associated antigens (TAAs) and prime adaptive immune cells (mainly T cells) against them. This leads to both: direct and indirect cancer killing, anticancer effector functions, production of anti-TAA antibodies and subsequent immunity capable of rejecting tumor cells possessing the corresponding TAAs (3, 8). In this complex interplay, one may appreciate that the step of “priming” adaptive immune cells by innate immune cells against TAAs represents a crucial milestone that is completely dependent on the antigen-presenting and antigen-sensing capabilities of innate immune cells (2). While most innate immune cells (professional presenters) and certain cells of epithelial lineage (non-professional presenters) are capable of presenting antigens to the adaptive immune cells (6) be it to varying degrees; yet the sentinel antigen-presenting cells (APCs) of the immune system are the DCs (2, 3, 9). DCs are the guardian APCs because they are both efficient at antigen-presenting and adaptive immune cell activation and also good at judging whether an entity possesses “self” or “non-self” antigens (2, 10, 11). The ability of DCs to present “non-self” TAAs properly to prime as well as to activate adaptive immune cells is an absolute pre-requisite for activation of potent anticancer immunity (2, 4).

In the present review we briefly discuss the basic biology of DC activation states that can make a difference between pro-tumorigenic inflammation and anti-tumorigenic immunity. We will then discuss in more detail the ability of anticancer therapeutics to influence and modulate these activation states and the crucial impact of exosomal communication on DC-associated functions.

## Dendritic Cells and Their Activation States: A Bird’s Eye-View

The molecular cell biology of DCs has evolved in a sophisticated manner to facilitate its APC functions (12). DCs in general possess a diverse repertoire of surface receptors (and intracellular receptors) that help them in environmental sensing and to carry out “at will” rapid innate immunity-related functions (2, 12). Such receptors include various scavenging or phagocytic receptors like CD91, integrins, CD36 (aiding in phagocytosis and clearance of target entities), surface pattern recognition receptors (PRRs) like toll-like receptors (TLRs), and intracellular PRRs like NOD-like receptors (NLRs) (10, 13, 14). DC-based PRRs help in detection (and subsequent DC stimulation) of danger signals like pathogen-associated molecular patterns (PAMPs) or damage-associated molecular patterns (DAMPs) (4, 5, 8).

Dendritic cells are also special in terms of their antigen processing machinery. Classically (for non-professional APCs and normal cells, as applicable), antigens derived from intracellular sources are presented by the major histocompatibility complex (MHC) class I presentation system while extracellular antigens (captured *via* phagocytosis or pinocytosis) are preferentially processed for MHC class II presentation (15). In specialized APCs like DCs however, the extracellular antigens can also gain access to the MHC class I presentation system (mediated by following events: phagophore → endosome → antigen escape from endosome → antigen processing by cytosolic proteasome for MHC I presentation) while intracellular antigen fragments can also be found on the MHC class II molecules (mediated by autophagy) – a phenomenon termed as “cross-presentation” (15). This unique ability to cross-present antigens to adaptive immune cells is also behind DCs’ significant role as APCs. Depending on the environment they encounter (e.g., normal “self” antigen rich environment or abnormal “non-self” antigen rich environment); DCs can exhibit various states and accordingly perform different functions (2, 12). Based on a highly stark difference between antigenic environments, i.e., host “self” antigens vs. foreign or pathogen-associated “non-self” antigens, DCs can exist in two main states, i.e., steady state immature dendritic cells (iDCs) and fully mature DCs (9, 12). The distinction between immature and mature DCs is partly based on changes occurring on two crucial levels, i.e., phenotypic level and functional level (2, 14, 16). Phenotypic maturation is attained when DCs up-regulate surface maturation ligands such as CD80, CD83, and CD86 along with the MHC class II molecule (9). DCs stimulated on the functional level exhibit the ability to secrete cytokines where the balance between inflammatory or immunostimulatory cytokines (e.g., IL-12, IL-6, IL-1β) and immunosuppressive cytokines (e.g., IL-10, TGF-β) is decided by the “environmental context” (2, 9, 17).

In normal, healthy conditions, DCs exist in an immature or steady state such that in this scenario their main aim is to maintain immune tolerance by impeding adaptive immune cells from attacking host cells that possess “self” antigens (4, 10, 12). However, if DCs encounter “non-self” entities in the periphery, they opsonize them, process their antigens for cross-presentation, migrate to the lymph nodes, and prime naïve T cells for these antigen (9). DCs provide the T cells with the information about whether an antigen is present and whether it poses a threat – a foundational mechanism for the subsequent T cell effector function (18). A single DC can contact as many as ∼5000 T cells per hour (19). Steady state iDCs exhibit continuous endocytic activity (20) and hence continuously present “self” antigens to T cells. However in this case the T cells are not polarized toward an effector state but are rather polarized to facilitate tolerance or immunosuppression (12, 21). Such immunotolerance is actively induced and maintained through a mixture of immune checkpoint pathways and complete lack of stimulatory signals provided by the DCs (22). Immune checkpoint pathways are a plethora of inhibitory cascades that are crucial for maintaining self-tolerance and modulation of duration/amplitude of immune response, e.g., DC-based presentation of ligands like cytotoxic T-lymphocyte-associated antigen 4 (CTLA4) and programed cell death protein 1 (PD1) to T cells causing T cell anergy or differentiation of immunosuppressive T cells (22). Such immunosuppressive T cells (e.g., regulatory T cells, T_regs_) further help in spreading tolerance toward “self-antigens” (6, 9). On the other hand, when DCs encounter pathogens or entities possessing PAMPs (detected in part through PRRs) they switch to a mature state exhibiting strong phenotypic and functional stimulation. At this stage, the DCs leave the function of phagocytic scavenging and assume the more sophisticated APC-function (12). Subsequently, DCs carefully co-ordinate their proteolytic processes in the cytosol (e.g., proteasomes), endosomes-lysosomes (e.g., lysosomal hydrolases), and the endoplasmic reticulum (ER) to degrade “non-self” entity-derived proteins in order to yield suitable antigenic peptides that are subsequently loaded on MHC class I and II molecules for presentation to T and B cells (9, 12). The simultaneous presence of phenotypic maturation ligands, suitable cytokines, other functional immunostimulatory factors, and appropriate antigen-MHC complexes helps activate an effector profile in interacting T cells thereby polarizing them for antigen-specific elimination of the “non-self” entity (9). Here, antigen-MHC complexes are the main stimulatory signals (signal 1, detected by the T cells through a complex of T-cell receptors/TCRs-CD3) followed by phenotypic maturation ligands. These ligands help in providing proper co-stimulation by binding corresponding receptors on T cells (signal 2, detected by T cell receptors like CD28, CD40L) in the presence of cytokines or factors eliciting immunostimulation and the effector T cell phenotype (signal 3, detected by respective cytokine cognate receptors) (9). The presence of these three signals is absolutely essential for effective T cell stimulation by APCs (like DCs) and their polarization toward anti-pathogenic effector function (6, 9). It is noteworthy though, that apart from these three signals, DCs might modulate T cell function via other immunomodulatory signals (e.g., exosomes, discussed later); however because they still lack a well-characterized functional status, they cannot yet be ascribed as *bona fide* T cell modulatory signals. Last but not least, it is important to consider that maturation of DCs is primarily crucial for the activation and differentiation of naïve T cells (10). Pre-existing cytotoxic T cells and memory T cell populations are not very strongly dependent on fully mature DCs for their effector functions (2, 3, 12).

## Tumor-Infiltrating DCs: An Overview

The Dichotomy of DC maturation states is mainly applicable to an environment where a very obvious distinction exists between “self” and “non-self” antigens. The continuum of DC activation states is much more complex when it comes to cancer as most cancerous tissues or tumors are very similar in terms of antigenic make-up to that of normal cells (5, 12). This is attributable to the fact that most antigens are either shared with nearby normal tissues (e.g., differentiation antigens) or with spatiotemporally distinct yet normal tissue [e.g., oncofetal antigens or cancer-testis antigens (7)]. This leads to a strong conflict regarding what represents “self” or “non-self” – which is further revived by the struggle between the tolerance-encouraging tendency of DCs and their propensity to prime T cells for tumor rejection (4, 9, 12). This situation is further exacerbated by the capacity of cancer cells to interfere with normal DC function (23) *via* immunosuppressive cytokines or other signals like those conveyed by exosomes (discussed later).

**Figure 1:**
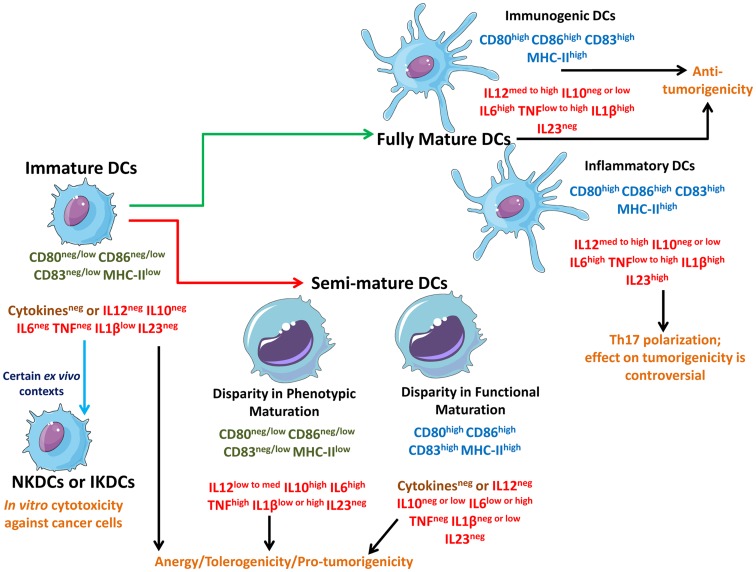
**Schematic representation of different states of DCs interacting with different types of cancer cells**. Live cancer cells and cancer cells undergoing non-immunogenic cell death keep the steady state DCs in an immature state devoid of strong up-regulation of phenotypic maturation ligands (CD80, CD86, CD83, MHC-II) and functional maturation (no or negligible amounts of immunostimulatory cytokines). In certain *ex vivo* conditions, immature DCs can behave like natural killer DCs (NKDCs) or interferon-producing killer DCs (IKDCs), which can exert *in vitro* anticancer cytotoxicity. On the other hand, freeze/thawing of cancer cells, certain immunogenic live cancer cells, and certain therapy-induced non-immunogenic cell death routines can induce a “limbo” state in DCs called semi-mature DCs which are not fully mature and can be either devoid of phenotypic maturation ligands or functional maturation depending on the context. Both immature DCs and semi-mature DCs cause T cell anergy and facilitate tolerogenicity thereby compromising anticancer immunity. These DCs may also actively facilitate pro-tumorigenic signaling. However, some therapeutic paradigms can induce immunogenic cell death (ICD) or at least a certain level of augmented immunogenicity in cancer cells which causes the interacting DCs to fully mature. These fully mature DCs can potently elicit anticancer immunity. Fully mature DCs devoid of immunosuppressive cytokines like IL-10 can be termed as immunogenic DCs capable of forming the most productive interface with T cells to prime them for anticancer effector function. On the other hand, fully mature DCs secreting IL-23 (inflammatory DCs) may polarize the T cells toward a state where they have a “helper” behavior accompanied by IL-17 production (Th17). The role of Th17 cells in cancer immunity and progression is enigmatic and controversial.

In a well-established tumor, cancer cells actively suppress steady state DCs (also called tumor-infiltrating DCs or tumor-infiltrating dendritic cell, TIDCs) and keep them in the favorable immature state (Figure 1) (23–25). Such immature TIDCs tend to exhibit dysfunction in antigen-presenting capabilities, suppressed endocytic activity, abnormal motility, and various other immature characteristics – a point that has been demonstrated in a number of studies analyzing various solid tumors and tumor-draining lymph nodes (26). Such induction of immature state in TIDCs by the tumor is not surprising considering that mature DC’s density in tumors inversely correlates with tumor pathologic grade/stage and positively correlates with improved prognosis (26). Moreover, tumors may also actively induce apoptosis in TIDCs through certain gangliosides (e.g., GM3, GD3), glycoproteins (e.g., MUC2 mucins), and neuropeptides (25, 26).

The tumor-induced iDCs state is mainly characterized by: (1) the total absence or presence of negligible amounts of well-processed cancer antigens (compromised signal 1 generation), (2) absence or trivial amounts of phenotypic maturation ligands or co-stimulatory molecules (ablation of signal 2), and (3) either complete absence or minor presence of functional stimulus/immunostimulatory cytokines like IL-12 (ablated signal 3) (7, 10, 12, 23). Such iDCs can also be encouraged by the presence of non-immunogenic cancer cell death [e.g., tolerogenic apoptosis (11)] (5, 21, 27, 28). The presence of signal 1, i.e., processed cancer antigens is very crucial for potent elicitation of anti-tumor immunity since signals 2/3 have less meaning in absence of signal 1 (18). Thus not surprisingly, one of the immunoevasive strategies employed by cancer cells is the down-regulation or loss of antigens (7, 21). DCs prime the T cells for cancer antigens in the lymph nodes in three phases (18, 29); Phase I lasts for ∼8 h and consists of transient interactions between T cells and antigen-presenting DCs (29). T cells integrate antigenic stimulus from several such Phase I encounters until the cumulative signal triggers the onset of Phase II. During Phase II (which lasts ∼12 h), T cells form a long-lasting stable contact with a single DC (29). It is noteworthy that this Phase I–II transition depends strongly on the concentration of antigenic peptide-MHC complex per DC (18, 30); higher the concentration, the faster the tendency of T cells to exit Phase I and reach Phase II (18). Thus, lower cancer cell-associated antigen levels make it harder for the T cells to exit Phase I – a scenario that leads to unstable DC–T cell interactions and compromised T cell immunity. Phase II is also the stage where T cells are further activated *via* DC-based signals 2 and 3 (29). Thereafter, the T cells enter Phase III during which they proliferate vigorously and return to short interactions with the DCs (29). It should be note however, that the above “three phase theory” of DC–T cell interactions is mainly based on *in vitro*/*ex vivo* studies using either model antigens or high concentrations of TAA-based immunodominant peptides. Such studies need to be extended to settings of DC–T cell interactions within a tumor-bearing host, in near future.

Apart from antigen down-regulation, cancer cells also directly induce an immature TIDC state through secretion of immunosuppressive factors like IL-10, VEGF, TGF-β, and PGE_2_ (7, 25, 27); thereby further compromising stable DC–T cell interactions. The strategies and mechanisms employed by cancer cells for inducing DC-based tolerogenicity have been discussed in details in certain recent reviews (5–7, 21). Curiously it has been demonstrated recurrently that in an *ex vivo* set-up, certain iDCs may exhibit the ability to directly lyse transformed cells or tumor cells *in vitro* (31). Such iDCs have been termed as natural killer dendritic cells (NKDCs) or more recently interferon-producing killer DCs (IKDCs) (Figure 1) (31) and have been found to exert anticancer cytotoxic activity *in vitro* in both rodent and human set-ups (31–33). While, IKDCs may simply reflect the prevalent *ex vivo* DC heterogeneity yet their characterization raises the need to better study DC features in tumor-bearing hosts.

## DC Activation States in Tumor Immunosurveillance and Anti-Tumor Immunity

As per the theory of cancer immunoediting, during tumor development the equilibrium between growing tumor and immune system shifts: at the beginning the immune system is capable of recognizing and exterminating cancer cells (“elimination” phase). Later, cancer “immunoediting” and release of cancer-derived immunosuppressive factors, results in the establishment of an equilibrium between cancer cells that are still susceptible to immunoeradication and immunoevasive ones that are resistant to anticancer immunity (“equilibrium” phase). Finally, as the immune evasion process progresses, the tumor escapes immune cell control (“escape” phase) (34). It has been long proposed that anticancer therapies should kill the cancer cells in a manner that helps activate the DCs to prime the adaptive immune system for anticancer activity (28, 35), however the experimental as well as clinical translation of this idea have unfortunately not been straightforward. This may result from the fact that most anticancer therapies tend to induce either non-immunogenic or very low-immunogenic cancer cell death (11) and thereby disallowing sufficient DC stimulation (5, 21, 27, 35) and keeps the DCs in an immature state (Figure 1). For instance, certain therapeutic modalities (e.g., chemotherapeutics like cisplatin) or certain anti-tumor vaccine-preparation methodologies (i.e., freeze/thawing, discussed later in Anticancer Therapy Differently Shapes the DC-Dying Cancer Cells Interface), may actually cause a sub-optimal activation of DCs (24, 28, 36, 37) thereby giving rise to a somewhat “limbo” state which can be termed as “semi-mature” DCs (Figure 1) (10). It is noteworthy though that in certain instances, semi-mature DCs generated *ex vivo* and injected back into the host (in this case rhesus macaque) might become mature spontaneously during migration before reaching the lymph nodes (38). However, whether this situation applies to therapeutic DC vaccines is an enigmatic question since the above mentioned study was not done within the context of anticancer DC vaccines. In various anticancer therapy settings (see Table 1 and Anticancer Therapy Differently Shapes the DC-Dying Cancer Cells Interface), DCs interacting with dead/dying cancer cells (treated with non-immunogenic or low-immunogenic anticancer agents) may attain a semi-mature state, i.e., while they may present low/medium levels of cancer antigens yet they either lack co-stimulatory signals (e.g., CD86) or suitable immunostimulatory cytokines (e.g., IL-12) (6, 10, 28, 37). Thus, semi-mature DCs, unlike iDCs, exhibit the ability to sustain at least two (i.e., signal 1 and either one of the other two signals) of the three signals required for successful/optimal T cell activation (23) but unfortunately not all three at once and thereby they exhibit an unstable interface with T cells that leads to active ablation of anticancer immunity (10) and clonal T cell anergy (20, 23, 24). Semi-mature DCs might exhibit inconsistency in either up-regulation of phenotypic maturation ligands or in secretion of cytokines (Figure 1). Semi-mature DCs with disparity in phenotypic maturation are able to secrete one or more of the few assorted cytokines like IL-10, IL-6, IL-1β, and tumor necrosis factor (TNF), but do so to a highly variable degree (in terms of amount and simultaneous presence of these cytokines together) (23, 28, 37). It is also noteworthy that certain well-established tumors composed of immunogenic cancer cells (e.g., melanoma) may also encourage formation of *de novo* semi-mature TIDCs rather than immature TIDCs due to the particular tumor microenvironment they can create (39). Together iDCs and semi-mature DCs tend to encourage T cell anergy or T cell exhaustion (9, 10), tolerogenicity toward the cancer cell (9, 31), and even active pro-tumorigenic activity (e.g., semi-mature DC-derived IL-6 may act as a growth factor for tumors expressing IL-6R-gp130 cognate receptors and/or IL-10 can act as a general immunosuppressor) (17, 40, 41).

**Table 1:** **Inducers of cancer cell death that stimulates full maturation of DCs**.

Anticancer therapy	*In vitro* Phagocytosis	Phenotypic maturation of DCs	Release of cytokines by DCs	Stimulation of T cells	*In vivo* mice experiments	Clinical data
Hypericin-PDT	Garg et al. (37)	Garg et al. (37)	IL-1β (37); IL-6 (50); NO (37); IL-12p70 (Dudek et al., unpublished data)	Proliferation (50) IFNγ release (50)	*In vitro*-treated cancer cells induce antitumor immunity in mice vaccination experiment (37)	
UVB	Kotera et al. (52)		IL-12 (52)		Pulsed-DC induce antitumor immunity in mice vaccination experiment (52)	
Cyclophospha mide (MAFO for *in vitro* experiments)		Kotera et al. (52), Schiavoni et al. (53)	IL-1β, IL-6, IL-12 (52, 53)			Increased infiltration of phenotypically mature DCs (53, 55–57); increased DCs trafficking to the lymph node (53)
γ Irradiation	Prasad et al. (58), Kim et al. (59)	Prasad et al. (58), Kim et al. (59)	IL-6 (59)	IFNγ release (62)	After tumor irradiation: (1) increase in tumor-infiltrating mature DCs (60, 61); (2) increase in IFNγ production by spleen cells (63)	
Doxorubicin	Obeid et al. (132)	Ghiringhelli et al. (134)	IL-1β (134)	Proliferation and IFNγ release (133)		
Oxaliplatin		Ghiringhelli et al. (134)	IL-12p70 (134)	Proliferation and IFNγ release (134)		
Bortezomib		Cirone et al. (135)		IFNγ release (135)	Pulsed-DC induce antitumor in mice vaccination experiment (136)	
CMQ and colchicines		Wen et al. (137)		Proliferation (137)		
Oncolytic viruses		Moehler et al. (73), Donnelly et al. (138)		Release of IFNγ (138), release of TNF and IL-6 (73)		

Recently however, it was described that certain therapeutic modalities [e.g., mitoxantrone/doxorubicin, hypericin-based photodynamic therapy (Hyp-PDT), and radiotherapy] cause cancer cells to undergo immunogenic cell death (ICD) (28, 35, 40, 42). ICD tends to be highly immunostimulatory because it emits a spatiotemporally defined combination of potent DAMPs that act as danger signals important for DCs stimulation (35). DCs detect such danger signals through a combination of receptors including TLRs, CD91, and purinergic receptors (21, 35). ICD may also ablate the canonical strategies harnessed by cancer cells to encourage the formation of immature or semi-mature DC states (21, 27). Beyond ICD, some anticancer therapeutics (e.g., antimitotic chemotherapeutics like docetaxel) may induce a general augmentation of immunogenicity that is not as strong as ICD but is still effective in a context-dependent fashion (43). Cancer cells undergoing ICD, or exhibiting therapy-induced (minor to medium increase of) immunogenicity, encourage the formation of fully mature DCs (Figure 1) (10, 27, 28, 35, 37, 43). In general, fully mature DCs exhibit all three conventional T cell stimulatory signals, thereby enabling elicitation of potent anticancer immunity (12, 13, 31). However, based on the pattern of only a few cytokines fully mature DCs might be subdivided, i.e., immunogenic DCs and inflammatory DCs (Figure 1) (35, 44). The fully mature immunogenic DCs are supposed to exhibit the least or total absence of immunosuppressive cytokines like IL-10 (17, 21, 40). Most known ICD inducers result in the formation of general fully mature DCs, with a context-dependent absence or reduced abundance of immunosuppressive cytokines (e.g., IL-10) (28, 37, 45). On the other hand, the presence of high IL-23 cytokine expression might be a marker of inflammatory DCs (44). Indeed, IL-23 may encourage T cells to exhibit the Th17 polarization (T helper cells/Th cells producing IL-17 cytokine) (44). It is noteworthy that the role of inflammatory DC-Th17 arc in cancer progression is still enigmatic with evidence supporting both anti-tumorigenic and pro-tumorigenic roles for this interaction, depending on the context (44, 46, 47). Thus for anticancer immunity, the functional role of fully mature inflammatory DCs needs to be treated with caution until further research ascertains their exact behavior.

It is noteworthy though, that the distinctions between different DC maturation or activation states made on the basis of phenotypic maturation markers or cytokine patterns are primarily based on *ex vivo* or *in vitro* experiments. This is because simultaneous analysis of various surface-associated and soluble DC activation markers is relatively easy *ex vivo* or *in vitro*. However, *in vivo* or *in situ*, such a simultaneous detection is nearly impossible. *In vivo* or *in situ*, mostly only the phenotypic maturation status of tumor-infiltrating DCs is detected *via* immunofluorescence staining (e.g., CD11b^+^CD11c^+^CD86^high^MHC-II^high^ DCs). While an analysis of cytokines associated with the tumor is possible via RT-PCR, proteomics-approaches, or antibody arrays, yet there is no way of characterizing which cytokines are secreted exclusively by the TIDCs. In future, lineage-tracing of the DCs in tumors or high enumeration staining/detection strategies for TIDCs might make it possible to simultaneously detect the phenotypic and functional markers of DCs *in vivo* or *in situ* however until that point, the above mentioned distinctions can be treated as operational definitions. Furthermore, it would be necessary to further characterize the additional states of semi-mature or fully mature DCs relevant for cancer treatment, not only *in vitro*/*ex vivo* but also *in vivo*/*in situ*.

## Anticancer Therapy Differently Shapes the DC-Dying Cancer Cells Interface

Anticancer therapies are capable of modulating DC states, either directly or *via* dying cancer cells. We believe that efficient anticancer treatment should be able to re-establish the recognition of cancer cells by the immune system, as well as “revive” the dominance of the immune system in this cross-talk. Therefore, the maturation status of DCs, as the predominant APCs, after anticancer therapy or after co-incubation with *in vitro*-treated dying cancer cells is an attractive marker of stimulation of an immune response, specifically relying on effector CD4^+^/CD8^+^ T cells (characterized by increased T cell proliferation/infiltration and secretion of IFNγ) (48).

Interestingly, cancer cells treated with most anticancer therapies either induce full DC maturation (a very small fraction of therapies) or do not stimulate the DCs at all (i.e., immature or tolerogenic DC formation, induced by a large fraction of therapies). There are however, a limited number of therapies that can also induce the formation of semi-mature DCs. In the next section, the formation of fully mature and semi-mature DCs will be discussed within the context of anticancer therapies.

### Fully mature DCs

Only few therapies have been reported to have the capability to induce cancer cell death that stimulates complete DC maturation. By complete maturation of DCs we understand induction of both, phenotypic markers and production of immune-stimulating cytokines. Instead, to the best of our knowledge, in most *in vitro* studies, the analysis of cytokine expression profile is either incomplete, or the most important cytokines, e.g., IL-12p70, IL-10, are not included. Only such fully mature DCs are able to stimulate T cells, hereby increasing their proliferation and secretion of IFNγ, which are often considered to be surrogate indicators of a productive immune stimulation. Thereby, in the absence of information on the full pattern of cytokines released by DCs’, an increase in T cell stimulation can be considered a strong indicator of a full maturation state of the aforementioned DCs. Moreover, full maturation of DCs can be assumed with high probability if anticancer immunity in syngenic mice vaccination models (e.g., B16 cells in C57Bl/6 mice, MCA205 cells in C57Bl/6 mice, CT26 cells in BALB/c mice, 67NR cells in BALB/c mice) is achieved when dying cancer cells, following chemotherapy *in vitro*, are administered (either in a prophylactic or curative set-up). An anticancer treatment that can induce productive maturation of dying cancer cell-loaded DCs, at least *in vitro*, is Hyp-PDT (37, 49–51). Already for some time it is known that Hyp-PDT-treated cancer cells induce both phenotypic and functional maturation of DCs (37, 50) and that in mice vaccination experiments, the dying cancer cells stimulate anticancer immunity preventing growth of transplantable tumors (37). Recently this data was re-confirmed and extended further (50). DCs interacting with Hyp-PDT-treated cancer cells exhibit a fully mature immunogenic phenotype functionally characterized by significant secretion of immunostimulatory factors like IL-1β, IL-6, nitric oxide, and the absence of the immunosuppressive cytokine, IL-10 (50). Moreover, Hyp-PDT-treated cancer cells elicit secretion of IL-12p70 by loaded DCs (Dudek et al., unpublished data).

Other treatments for which the detailed immune-effects have been described include UVB irradiation, cyclophosphamide, and γ-irradiation. There is evidence that UVB-induced dying cancer cells are phagocytosed by DCs, leading to an increase in IL-12 production (52). Furthermore, DCs pulsed with UVB-treated B16F10 cells, induce anti-tumor immunity in mice and prevent growth of transplantable tumors (52). As cyclophosphamide requires hepatic activation, for *in vitro* investigations its analog, MAFO, is used. Exposure of DCs to mafosfamide (MAFO)-treated cancer cells causes phenotypic maturation of DCs and their functional stimulation, characterized by the release of various cytokines (IL-1β, IL-6, IL-12) (53, 54). Moreover, the treatment with cyclophosphamide of tumor-bearing mice results in increased tumor bed infiltration by phenotypically mature DCs (53, 55–57), as well as increased trafficking of DCs from the tumor bed to the draining lymph nodes (53). Furthermore, cyclophosphamide, when given to patients at metronomic doses, combines direct effects on immune cells, like: limitation of T_reg_ cells population and expansion of DCs in peripheral blood (56, 57) with potent stimulation of a DC response. Also γ-irradiated murine melanoma cells are efficiently phagocytosed by DCs, resulting in their phenotypic maturation (58, 59). Despite the fact that neither IL-12p70 nor TNF are secreted by loaded DCs, these cells release another pro-inflammatory cytokine, IL-6 (59). These observations prove the triggering of a functional, however not optimal (lack of IL-12p70), maturation of loaded DCs. The positive immunostimulatory effects of γ-irradiation were shown by increased tumor-infiltrating active DCs following local high-dose radiotherapy (60, 61). Furthermore, when human monocyte-derived DCs and irradiated melanoma cells were co-incubated with T cells, T cell-derived IFNγ secretion increased (62), an observation that was also substantiated *in vivo* when irradiation of established B16F10 tumors resulted in an increase of IFNγ-producing spleen cells (63).

However, as mentioned, complete analysis of the effects of drug-treated cancer cells on DC maturation is limited to only few therapies. Other treatments are simply hypothesized or speculated to induce fully mature DC phenotype, but these are claims supported by only indirect data. Table 1, recapitulates the available information about DCs-stimulating capacities of anticancer treatments.

Besides these conventional/experimental anticancer treatments, it is also emerging that targeted therapies can induce cancer cell death, capable of affecting DC maturation status. One such therapy is Vemurafenib (PLX4032), the inhibitor of mutated BRAF^V600E^ kinase, which is predominantly used in patients with melanoma. Incubation of cancer cells (that harbor BRAF^V600E^ mutation) with iDCs followed by poly(I:C) stimulation of the latter, down-regulated the release of TNF and IL-12 (IL-12 being crucial for effective functional maturation of DCs) (64). However, when cancer cells were pre-treated with Vemurafenib, the release of TNF and IL-12 from poly(I:C) matured DCs was re-established to a level obtained in the control (matured DCs without cancer cells) (64). Moreover Vemurafenib is known to increase TAA levels, such as MART1 and gp100 (65).

In conclusion, in future it is necessary to find and test more ICD inducers in order to better understand the diversity that fully mature DCs may exhibit in terms of activation characteristics. Also, it would be necessary to (re-)analyze certain existing therapies for their potential to cause DC maturation irrespective of whether they induced ICD.

### Semi-mature DCs

In the literature, evidence indicates that some anticancer treatments may cause “moderate” stimulation of an immune response. Under such circumstances the immune system activating signals are not strong enough or not persistent enough to establish a stable anticancer immunity. For DCs, this means that these APCs lack either the required phenotypic maturation markers and thereby are not capable to efficiently interact with T cells, or the required signature cytokine pattern released from loaded DCs and ultimately resulting in “immunocompromising” actions. Tolerogenicity induced by semi-mature DCs is connected with release of immunosuppressive cytokines like IL-10, TGF-β (66), plasma membrane expression of programed cell death ligands, like PD-L1 or PD-L2 (67), and with stimulation of T_regs_ expansion (67).

#### Phenotypically mature DCs

A good example of a treatment that induces phenotypic maturation of DCs, independent of the immunostimulating profile of cytokines is bevacizumab. This epidermal growth factor receptor (EGFR)-blocking antibody, which blocks angiogenesis, only induced phenotypic maturation of DCs upon their co-incubation with treated cancer cells (68). Nevertheless it should be highlighted that, on the one hand, addition of bevacizumab to co-cultures resulted in increased IL-6, but decreased IL-12 release (68). Moreover, it was shown that bevacizumab-treatment of patients with metastatic colorectal cancer increased total lymphocyte number (69) and had the potential to increase extravasation of T cells into the tumor bed, previously observed for the therapeutic paradigm of anti-EGFR antibody combined with adoptively transferred T cells in mice models (70).

Furthermore, cetuxinib, another EGFR-blocking antibody that prevents signaling from growth factors, shows similar results. Despite the fact that colon cancer cells treated *in vitro* with cetuxinib were phagocytosed by iDC (71) and induced the up-regulation of maturation markers (72), there is no investigation, till now, of the cytokines required for characterization of maturation status of these DCs. Thus this treatment should not be incorporated into the group of therapies that induce “full mature DCs” – as of now. Nevertheless, cetuxinib treatment has other features that demonstrate its positive effect on the immune system: its capacity to stimulate NK cell mediated antibody-dependent cellular cytotoxicity and complement-dependent cytotoxicity, are well documented (72).

Finally sunitinib (an inhibitor of receptor tyrosine kinase)-treated melanoma cells enhanced the maturation status of DCs (measured by the percentage of CD86^+^ cells). However no investigation of DC-secreted cytokines has been performed. In spite of this, one can be relatively positive about its immunoinhibitory effects, as cytotoxic T lymphocytes (CTLs) incubated with these loaded DCs did not increase their secretion of IL-6 (73).

Thus, while in some cases, certain therapeutic treated cancer cells induce formation of semi-mature DCs, yet for others the indications in this direction are either mixed or poorly studied. More analysis is required on the cytokine levels to ascertain whether such therapeutics are able to cause formation of semi-mature DCs or not. Last but not least, it is also necessary to analyze further the direct effects of anticancer treatments on DCs maturation (in set-ups where these therapies are not intended to directly affect the DCs) – an aspect that has received the least attention in studies addressing DC-based immunity.

## DC-Based Cancer Immunotherapy

Most of the clinically used anticancer therapies if systemically administered strongly affect not only cancer cells but as well the cells from tumor microenvironment, systemic hematopoietic cells, and rapidly dividing bone marrow cells. Despite the fact that recently platinum-based drugs, at clinically applicable concentrations, have been shown to enhance cytokine-induced DC maturation *in vitro* (74), vast majority of the effects on the non-cancer cells are of negative nature (i.e., prevention of differentiation of new immune cells from progenitor bone marrow cells and lymphopenia or leukopenia). These actions reduce the number of immune cells capable of sensing the danger and immune-stimulating signals released by dying cancer cells thereby compromising anticancer immunity. To evade this effect, a DC-based immunotherapy approach can be employed in a couple of ways: (1) by directly targeting/stimulating the DCs *in vivo* so as to accentuate their anticancer phenotype or (2) by stimulating the DCs *ex vivo* and infusing them back into the host for carrying out anticancer effector function.

Starting from 1998 there were few trials testing the *in vivo* DCs’ stimulation with synthetic peptides (75–77). Most of them however failed as they were unable to effectively stimulate CD4^+^ cellular responses (75, 78, 79) and stimulation of Th2 type cytokines (80, 81). Learning from the abovementioned studies, Walter *et al*. showed that patients pre-treated with single-dose cyclophosphamide as well as vaccinated with TAAs peptides and granulocyte macrophage colony stimulating factor (GM-CSF), showed clinical responses in Phase I and II trials (82). To further improve the peptide/protein anticancer vaccines the idea of combining TLR agonist administration with the vaccines emerged. The idea was taken up by GlaxoSmithKline that invented AS15 adjuvant that combines TLR4 and TLR9 agonists (83). Patients with MAGE-A3^+^ melanoma administered with MAGE-A3 peptide in combination with AS15 in Phase II trial (NCT00086866 and NCT00290355) showed clinical activity (84). The study is being followed up by a Phase III trial.

An alternative, to direct *in vivo* DCs’ stimulation is, isolation of DCs’ precursors from the patient (through leukapheresis) and maturation/stimulation of these precursors *ex vivo* followed by allogeneic injection of these fully mature DCs back into the patient. Nowadays there are various ways applied to generate cancer cells-specific DCs: the stimulation can be done with specific TAAs (full length or short peptides), tumor lysates (freeze-thawed or acid eluted), electroporation/transfection of DCs with total cancer cell-mRNA, creating DC-cancer cell fusions, or with tumor derived exosomes (TDEs) (as discussed below). Alternatively DCs can also be genetically manipulated to express specific TAAs. Moreover as the stimulation is performed *ex vivo* there is a possibility to additionally co-stimulate with cytokine “cocktails” to assure their strong maturation. For example in 2010 a Provenge treatment strategy on similar lines got approved by FDA for therapy of patients with castration-resistant prostate cancer (85). The treatment consists of *ex vivo* stimulation of DCs with PA2024 that is a fusion protein of prostatic acid phosphatizes (TAA present in 95% of this type of tumor) and GM-CSF. The Phase III clinical trial revealed increased overall survival of patients treated with Provenge in comparison to placebo (86, 87).

Currently, there are many Phase I, II, and III clinical trials that test the effect of different anticancer DC vaccination strategies on various cancer types. The running/finalized clinical trials were recently thoroughly summarized by Galluzzi *et al*. thus we refer readers interested in this topic to “Trial Watch” publication (88).

## Exosomes; as Long Distance Messengers, Modulators, or Suppressors of DC-Associated Anticancer Immunity?

Phenotypic maturation and functional stimulation are well-established markers of DC maturation as well as the ability of DCs to “prime” anticancer immune responses (9). Modulation of these two relevant DC-associated biological parameters by cancer cells (on the levels of TAAs, DAMPs, or danger signals and cytokines/chemokines) is considered to make the difference between immature, semi-mature, and mature DCs (2, 7, 35, 40), as discussed above. However, depending on the anticancer therapy under consideration, DC markers and cancer cell-based modulators sometime fail to completely account for the observed failure of or reduction in anticancer immunity (89). Thereby these may point toward other DC or cancer cell-based autocrine or paracrine modulators of immunity which are capable of transmitting signals (21).

One vehicle type capable of long distant transport of cellular material are the endosome-derived nano-vesicles, known as exosomes (90). These vesicles are derived by inward budding of the multi-vesicular body membrane and have been implicated in cell-cell communication (91). Historically, exosomes were classified as a simple mechanism for the removal of unwanted cellular material (92, 93), yet more recently they have been implicated in the transmission of signals between cells, both locally and over long distances, effecting cells of different lineages, demonstrating the capacity to influence cellular signaling, and outcomes of stress responses (94–96). Where the physiological outcome depends on both the type of cell the exosome originates from and the type of stress the cell is exposed to, known to alter protein and lipid signatures in a context-dependent manner.

Certain cancer cells are known to exaggerate their generation of exosomes, demonstrating constitutive release, delivering tumor derived signals throughout the local tumor microenvironment and beyond within various body fluids (97, 98). These signals have been implicated in the transmission of pro-tumorigenic, angiogenic, and metastatic signals, as well as factors capable of stimulating/inhibiting immune responses (97, 99, 100). Here we will focus on the dynamic relationships that exist between the signals released or received between cancer cells and DCs and highlight key components that may sway the outcome in the context on anticancer immunity.

### The effect of cancer-derived exosomes on dendritic cells

Antigen acquisition by DCs is an essential step in the induction of antigen-mediated immune responses. These antigens can be sequestered by DCs in the form of infectious agents, dying infected cells or in the case of tumors, by the engulfment of dying cells or exosomes that are secreted by living/stressed or dying cells. As the protein signature of an exosome is dependent on the cell of origin as well as their viability, TDEs are abundant in TAAs (Her2/Neu, MART1, TRP1/2, gp100) (101), antigen-presenting molecules (MHC class I, heat-shock proteins) (102), as well as varying tetraspanins (such as CD81) (103–105). These privileged carriers of antigens and immunostimulatory molecules exposed on exosomes have been shown to activate DCs (101). Research identified that exosomes could induce phenotypic and functional maturation of DCs, demonstrating enhanced cell surface expression of MHC-II, CD80, CD86, and CD40 as well as increased IL-12p70 production (106). For example, melanoma exosomes were shown to deliver MART1 tumor antigens to monocyte-derived DCs, allowing for successful cross-presentation (101). Moreover, *in vivo* assessment of TDEs capacity for immunomodulation demonstrated their potential to prevent autologous tumor development, in a CD4/CD8-dependent manner (107). TDE mediated DC maturation and antigen presentation (MHC-II and ICAM) propagates T cell stimulation, demonstrated by increased CD4^+^ and CD8^+^ T-cell proliferation, the induction of enhanced CTL based tumor cell lysis (108, 109) and the generation of Th1-type memory (110). Moreover, exosomes derived from DC cells exposed to TAAs demonstrate 50-fold higher efficiency and 3-fold higher T cell activation potential than non-TAA exposed controls (109).

Conversely, other studies have demonstrated the immunosuppressive nature of TDEs. Work into the role of TDEs highlighted their tumor suppressor potential (111), however the majority of data, till now, indicates a more potent immunosuppressive nature. For example, TDEs can prevent DC differentiation *in vitro*, in such a manner that a pool of CD14^+^ HLA-DR^neg/low^ cells was generated, culminating in the marked reduction of autologous T cell stimulation (112). Also, *in vivo* experiments demonstrated an accumulation of undifferentiated myeloid cells in the spleen of mice after TDEs administration, consequently resulting in the formation of a DC population that was incapable of maturation (99). Furthermore, this inhibition of DC maturation/differentiation was also observed in human monocytes, following exposure to TDEs (99). Moreover, TDEs have the potential to activate myeloid derived suppressor cells (MDSCs), hampering immune responses, in this case *via* T_regs_ (113).

The potential for TDE to influence an immune response has generated contrasting bodies of research. However these observations may both be true and simply a consequence of experimental design. For instance, time is an important issue for response outcome when the TDE interact with immune cells. Yu *et al*. investigating the effect of TDE on bone marrow derived myeloid precursors, described a significant reduction in DC differentiation, induced by treatment with GM-CSF, when the exposure occurred within 3 days (99). In contrast, Andre *et al*. showed that pulsing iDCs with TDE, after 5 days of GM-CSF treatment, resulted in an observed DC-mediated T cell response (101). Moreover, research into the effect of TDE, on induction of cytokine release from monocytes, demonstrated that a cacophony of pro-inflammatory cytokines (such as TNF, IL-6), as well as immunosuppressive factors (such as IL-8, IL-10, TGF-β) (114) were released. Importantly immunosuppression was predominantly mediated *via* TGF-β. The ability of TDE to induce IL-6 expression and/or release has been implicated in their inhibition of myeloid precursor differentiation, as well as accentuating the immunosuppressive capacity of MDSC, which were themselves activated by TDEs (99). Furthermore, research demonstrated enhanced exosomal HSP72, induced by IFNγ stimulation of tumor cells, resulted in the up-regulation of CD83 and potentiation of IL-12 production in DCs (115).

Alterations in cancer dendritic cell-derived exosome (DEX) expression of key immune-modulators have been shown to be evoked by both tumor microenvironmental stress as well as cellular stress induced by anticancer therapies, both traditional and targeted approaches (101–105). However, due to the vast number of cell types that excrete exosomes, little is known about the effect of therapy specifically on exosome-based host immune activation in clinical settings. However, research *in vitro* has demonstrated significant enrichment of TAAs as a consequence of therapy. Moreover, combination therapy with exosomes and DNA alkylating agents (such as cyclophosphamide) significantly potentiated cancer killing compared to single agent (117, 118). Fortunately, due to the biomarker potential of exosomes, progress into exosomal population isolation is allowing further investigations of the immunomodulatory and overall clinical potential of TDEs (116).

Importantly, the mode of antigen secretion can also alter the immunogenicity toward TAAs (119). Antigens loaded into nano-vesicles were shown to incite a significantly stronger immune response, than when the same antigens were secreted freely. Therefore the manipulation of how antigens are presented to immune cells may be used to enhance the success of anti-tumor vaccinations (107). So, due to the contradictory effects of TDE on DC-induced immune responses, the concept of TDE as a targeted-cancer therapy was quickly surpassed by the use of safer and more focused DC-DEXs, loaded with TAAs (120, 121).

### The effect of dendritic cell-derived exosomes on cancer cells

The potential for endogenous DEXs to induce anticancer responses remains unclear. However, existing research has identified that DEXs express, on their surface, multiple TNF superfamily ligands (122). Through these ligands they can incite anticancer immunity directly *via* the induction of cancer cell apoptosis, as well as indirectly through the activation of NK cells (122, 123). Recent work shows that similar to DCs, DEXs contain TNF, FasL, and TRAIL. These ligands have the potential to trigger caspase activation and apoptosis in a tumor cell models (122). Moreover, DEXs can also activate NK cells and stimulate their IFNγ secretion, inciting immune responses (122, 123).

However, research over the past decade has highlighted more the use of engineered DEX as a feasible and successful route to activate anti-tumor modalities *in vivo* (123, 124), that has gone on to demonstrate success clinically (120). Interestingly, treatment with engineered DEXs has shown a stronger anticancer effect than the use of the DCs they are derived from to re-activate downstream immune responses. These observations may in part be explained by the immunosuppressive effect of the tumor microenvironment on DC phenotypic functionality (125). Zitvogel and colleagues demonstrated a perturbation in growth of mastocytoma and spontaneous mammary carcinoma tumors by day 10, following inoculation with DEXs, derived from bone marrow DC that were pulsed with acid eluted tumor antigens (107). Furthermore, by day 60 ∼50% of mice treated with DEXs were diagnosed tumor free (107). Interestingly, when re-challenged, the mice demonstrated tumor rejection unless inoculated with a differing cancer type, implying long-lasting anti-tumor immunity stimulated by DEXs (107). Furthermore, Taieb *et al*. investigating the combination of DEXs with cyclophosphamide showed that DEXs were capable of boosting the immune response toward immunogenic cancers, showing synergistic tumoricidial potency toward pre-established tumors (118).

Elegant research into the potential of DEXs as anticancer modulators demonstrated that DEXs harvested from bone marrow derived DCs that had been stimulated by LPS treatment mature dendritic cells derived exosomes (mDEXs), compared to untreated immature dendritic cells derived exosomes (imDEXs), were significantly enriched in molecules (such as ICAM-1) capable of mediating T cell priming, enhanced T cell proliferation and the stimulation of naïve T cells to differentiate and produce cytokines (108). The research of Naslund and colleagues showed that DEX treatment induced T cell responses, yet in a B cell-dependent manner (126). This suggests that immunization with DEXs carrying only peptides for T cells would induce a sub-optimal response (126). Furthermore, protein-loaded rather than peptide-loaded DEXs showed greater T cell responses *in vivo* and a superior anti-tumor capacity (126). Interestingly, the induced T cell response requires the presence of B cells and mice deficient in complement activation and antigen shuttling by B cells had reduced DEXs-induced responses (126). Solidifying the dynamics of exosomal signaling in immune cell activation and anti-tumor immunity, DEXs secreted into the extracellular milieu during cognate T cell–DC interactions, are targeted and engulfed specifically by T cells, via the leukocyte function-associated antigen-1 (LFA-1) receptor (127).

Moreover, findings from preliminary Phase I clinical trials for the use of DEXs as a treatment for stage IV melanoma and non-small cell lung cancer, demonstrated a restoration of NK cell activity in over 50% of patients (107, 120). This increase in NK cell activity was shown to stimulate their cell killing capacity *in vitro* (120). Therefore, their lipid composition, that itself possesses adjuvant qualities and exosome stability within the circulation (128–130), coupled with simultaneous expression of MHC class I and II molecules, as well as a plethora of co-stimulatory molecules (102, 131), may indicate the cocktail of requirements that deem DEXs capable to incite anti-tumor or pro-immunogenic effects. Furthermore, the reported lack of toxicity highlights DEX-based therapies as an interesting modality for cancer therapy (107, 120). Further to this, investigation on combination of DEX-targeted therapies with traditional therapeutics or other modern targeted approaches should be done to explore their potential to restore immune activity in the fight against cancer.

## Concluding Remarks

The induction of an efficient anticancer immune response is thought to contribute to the success of anticancer treatments, by the establishment of a robust T cell mediated response capable of sustaining long-term control of cancer. Upon activation, DCs are crucial inducers of T cell immunity and are therefore at the frontline of immune-regulated responses. Hence, triggering proper maturation of DCs is an outstanding therapeutic objective as it may boost anti-tumor immunity and thwart cancer-induced immunosuppression. The discovery of different DCs sub-populations that exhibit wide functional plasticity has made the initial dichotomy between immature/tolerogenic and mature/immunogenic DCs, obsolete. However, in spite of a functional definition of these DCs phenotypes, which ranges from tolerogenic, partial/semi-mature to fully mature DCs, it still remains challenging to understand how, when, and to what extent this dynamic spectrum of DC activation drives tumor-specific tolerance or anti-tumor immunity, also in the context of anticancer therapy. In this respect, the existing (mostly immunosuppressive) or therapy-generated tumor microenvironments and the cross-talk between (dying) cancer cells and DCs, established through soluble (cytokines/chemokines) and vesicular (exosomes) mediators, are emerging as crucial determinants of DC maturation status and anticancer immune responses. Future preclinical research combined with clinical investigations, will disclose whether therapeutics inducing immunogenic cancer cell death, will meet the therapeutic objective of re-establishing the proper interface between dying cancer cells and DCs, promoting their fully mature/immunogenic status that is required to sustain anti-tumor immunity.

## Conflict of Interest Statement

The authors declare that the research was conducted in the absence of any commercial or financial relationships that could be construed as a potential conflict of interest.

## Abbreviations

APC(s), antigen-presenting cell(s); CD, cluster of differentiation; DAMPs, damage-associated molecular patterns; DC(s), dendritic cell(s); DEX(s), dendritic cell-derived exosomes; ER, endoplasmic reticulum; GM-CSF, granulocyte macrophage colony stimulating factor; Hyp-PDT, hypericin-based photodynamic therapy; ICD, immunogenic cell death; iDC(s), immature dendritic cell(s); IFN, interferon; IKDCs, IFN-producing dendritic cells; IL, interleukin; imDEXs, immature dendritic cells derived exosome(s); LFA-1, leukocyte function-associated antigen-1; mDEXs, mature dendritic cells derived exosome(s); MDSC, myeloid derived suppressor cells; NK, natural killer cells; NKDCs, natural killer dendritic cells; NLRs, NOD-like receptors; PAMPs, pathogen-associated molecular pattern(s); PRRs, pattern recognition receptors; TAA(s), tumor-associated antigen(s); TDE(s), tumor derived exosome(s); TGF, transforming growth factor; TIDCs, tumor-infiltrating dendritic cells; TLRs, toll-like receptors; TNF, tumor necrosis factor.
